# Mutant IP_3_ receptors attenuate store-operated Ca^2+^ entry by destabilizing STIM–Orai interactions in *Drosophila* neurons

**DOI:** 10.1242/jcs.191585

**Published:** 2016-10-15

**Authors:** Sumita Chakraborty, Bipan K. Deb, Tetyana Chorna, Vera Konieczny, Colin W. Taylor, Gaiti Hasan

**Affiliations:** 1 National Centre for Biological Sciences, Tata Institute for Fundamental Research, Bellary Road, Bangalore 560065, India; 2 Department of Pharmacology, University of Cambridge, Tennis Court Road, Cambridge CB2 1PD, UK

**Keywords:** Ca^2+^ signalling, *Drosophila*, IP_3_ receptor, Orai, STIM, Store-operated Ca^2+^ entry

## Abstract

Store-operated Ca^2+^ entry (SOCE) occurs when loss of Ca^2+^ from the endoplasmic reticulum (ER) stimulates the Ca^2+^ sensor, STIM, to cluster and activate the plasma membrane Ca^2+^ channel Orai (encoded by *Olf186-F* in flies). Inositol 1,4,5-trisphosphate receptors (IP_3_Rs, which are encoded by a single gene in flies) are assumed to regulate SOCE solely by mediating ER Ca^2+^ release. We show that in *Drosophila* neurons, mutant IP_3_R attenuates SOCE evoked by depleting Ca^2+^ stores with thapsigargin. In normal neurons, store depletion caused STIM and the IP_3_R to accumulate near the plasma membrane, association of STIM with Orai, clustering of STIM and Orai at ER–plasma-membrane junctions and activation of SOCE. These responses were attenuated in neurons with mutant IP_3_Rs and were rescued by overexpression of STIM with Orai. We conclude that, after depletion of Ca^2+^ stores in *Drosophila*, translocation of the IP_3_R to ER–plasma-membrane junctions facilitates the coupling of STIM to Orai that leads to activation of SOCE.

## Abstract (precis)

**Summary:** In *Drosophila* neurons, mutant IP_3_ receptors disrupt store-operated Ca^2+^ entry by destabilizing interaction of STIM with the Ca^2+^ channel, Orai. The interactions could coordinate store emptying with Ca^2+^ entry.

## INTRODUCTION

Receptors that stimulate phospholipase C and, hence, formation of inositol 1,4,5-trisphosphate (IP_3_) typically evoke both release of Ca^2+^ from the endoplasmic reticulum (ER) through IP_3_ receptors (IP_3_Rs) and Ca^2+^ entry across the plasma membrane. The latter is usually mediated by store-operated Ca^2+^ entry (SOCE), an almost ubiquitously present pathway through which empty Ca^2+^ stores stimulate Ca^2+^ entry across the plasma membrane (Putney and Tomita, 2012). The core molecular components of SOCE are stromal interaction molecule (STIM) and Orai (the gene for which is also known as *Olf186-F* in flies) (Hogan, 2015; Lewis, 2012). Orai forms a hexameric Ca^2+^-selective ion channel in the plasma membrane (Hou et al., 2012) and STIM is the Ca^2+^ sensor anchored in ER membranes (Carrasco and Meyer, 2011). Ca^2+^ dissociates from the luminal EF-hand of STIM when Ca^2+^ is lost from the ER. This causes STIM to oligomerize, unmasking residues that interact with Orai, and allowing STIM to accumulate at ER–plasma-membrane junctions, where the gap between membranes is narrow enough to allow the cytosolic CAD region of STIM to bind directly to Orai (Hogan, 2015). That interaction traps STIM and Orai clusters within ER–plasma-membrane junctions and it stimulates opening of the Orai channel (Wu et al., 2014). Additional proteins also regulate SOCE, often by modulating interactions between STIM and Orai (Srikanth and Gwack, 2012) or by facilitating their interactions by stabilizing ER–plasma-membrane junctions (Cao et al., 2015) or the organization of phosphatidylinositol 4,5-bisphosphate (PIP_2_)-enriched membrane domains (Sharma et al., 2013).

SOCE can be activated by thapsigargin, which depletes Ca^2+^ stores by inhibiting the ER Ca^2+^ pump, but for SOCE evoked by physiological stimuli, the Ca^2+^ stores are depleted by activation of IP_3_Rs. In the present study, we use genetic manipulations in *Drosophila* neurons to ask whether IP_3_Rs regulate SOCE solely by mediating Ca^2+^ release from the ER or whether they can also play additional roles downstream of store depletion. *Drosophila* is well suited to this analysis because single genes encode IP_3_R, STIM and Orai, whereas vertebrates have several genes for each of these proteins. Our results demonstrate that in *Drosophila*, its IP_3_R contributes to assembly of the STIM–Orai complex. Comparison of results from *Drosophila* and vertebrates suggests that the STIM–Orai complex might assemble in plasma-membrane–ER regions equipped to allow local depletion of Ca^2+^ stores.

## RESULTS

### Mutant IP_3_Rs attenuate SOCE in *Drosophila*

**Fig. 1.**
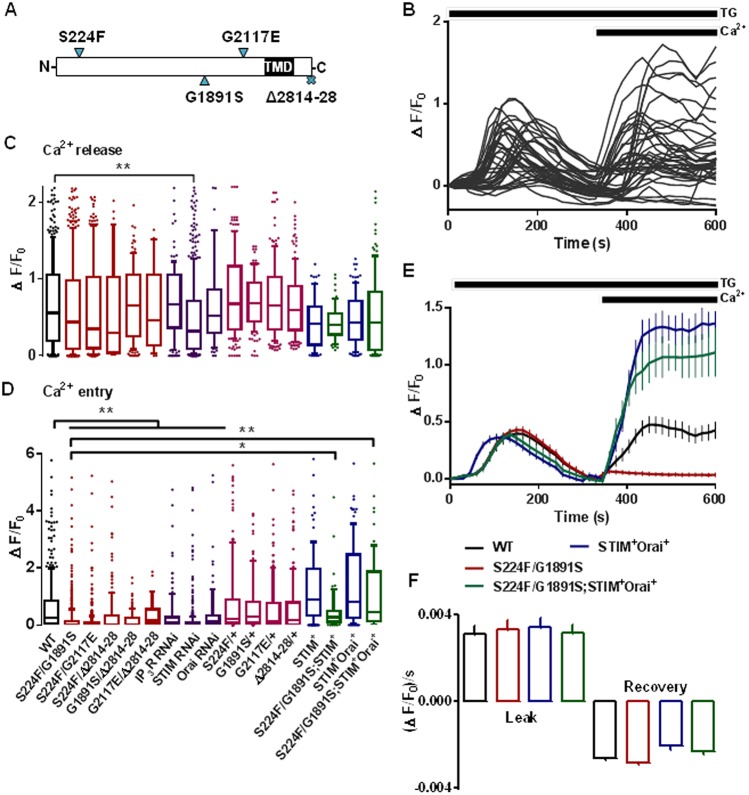
**SOCE in *Drosophila* neurons is attenuated by mutant IP_3_Rs and rescued by overexpression of STIM and Orai.** (A) IP_3_R mutations examined. TMD, transmembrane domains. (B) Traces from 40 individual wild-type (WT) neurons showing Ca^2+^ release evoked by thapsigargin (TG, 10 µM) in Ca^2+^-free HBM, and SOCE after restoration of extracellular Ca^2+^ (2 mM). (C,D) Summary results for peak responses evoked by thapsigargin (Ca^2+^ release) and Ca^2+^ restoration (SOCE) for neurons with the indicated genotypes and for WT neurons treated with the indicated siRNA. The box represents the 25–75th percentiles, and the median is indicated. The whiskers show the 10–90th percentiles. Outliers are represented by dots. Results are from >100 cells from at least five independent experiments.*P<0.05, **P<0.01 (Kruskal–Wallis test, followed by Wilcoxon signed-rank post-hoc test). (E) Responses of neurons from WT or *itpr* mutants (S224F/G1891S) alone and after overexpression of STIM and Orai. Results (mean±s.d., from >100 cells from at least five independent experiments) show Ca^2+^ release evoked by thapsigargin (10 µM) in Ca^2+^-free HBM, and SOCE evoked by subsequent addition of extracellular Ca^2+^ (2 mM). (F) Rates of Ca^2+^ leak and recovery from the thapsigargin-evoked Ca^2+^ release, calculated from E. The colour key applies to panels E and F. STIM^+^, overexpression of STIM; Orai^+^, overexpression of Orai.

SOCE evoked by depleting ER Ca^2+^ stores with thapsigargin in *Drosophila* neurons was abolished by RNA interference (RNAi) treatment for STIM or Orai (see Fig. 1D; Venkiteswaran and Hasan, 2009). This is consistent with evidence that STIM and Orai are core components of SOCE. Subsequent experiments examine the role of the IP_3_R, which is encoded by a single gene (*itpr*) in *Drosophila*, in regulating SOCE. To characterize SOCE in *Drosophila* neurons with mutant *itpr*, we examined five hetero-allelic combinations of a 15-residue C-terminal deletion and three point mutations located in different parts of the IP_3_R (Banerjee et al., 2004; Joshi et al., 2004) (Fig. 1A). We used these combinations because the adults with these mutations are viable with distinct flight phenotypes, whereas homozygotes and other hetero-allelic combinations are lethal (Joshi et al., 2004). We also used neurons heterozygous for each individual mutation. The peak Ca^2+^ signals evoked by addition of thapsigargin in Ca^2+^-free medium and the response to restoration of extracellular Ca^2+^ (SOCE) were measured in primary neuronal cultures for each genotype (Fig. 1B–D). Our use of fluo 4 fluorescence changes (ΔF/F_0_, see Materials and Methods) to report cytosolic free Ca^2+^ concentration ([Ca^2+^]_c_) is vindicated by evidence that [Ca^2+^]_c_ in unstimulated cells was unaffected by mutant IP_3_R (Fig. S1A) and the peak fluorescence changes evoked by SOCE in wild-type neurons were only 32±14% (mean±s.d., *n*=9) of those evoked by saturating the indicator with Ca^2+^.

SOCE was significantly reduced in all five *itpr* mutant combinations (Fig. 1D; Table S1). However, the resting [Ca^2+^]_c_ and thapsigargin-evoked release of Ca^2+^ from intracellular stores were unaffected, confirming that mutant IP_3_R selectively perturbed SOCE (Fig. 1C; Fig. S1A). The reduced SOCE in *itpr* mutant neurons was not, therefore, restricted to a single combination of mutant alleles. Mutant combinations in the ligand-binding domain (S224F), modulatory domain (G1891S and G2117E) and C-terminus (Δ2814–2828) of the IP_3_R all inhibited SOCE. SOCE was not significantly affected in neurons heterozygous for the individual mutants (Fig. 1D; Table S1). In adult flies, these heterozygous *itpr* mutants also have no significant effect on viability or flight phenotype (Banerjee et al., 2004; Joshi et al., 2004). The results establish that attenuated SOCE in *Drosophila* neurons is due to a perturbation of IP_3_R function in the recessive heteroallelic mutant combinations. This conclusion is supported by evidence that RNAi-mediated knockdown of IP_3_R also inhibited thapsigargin-evoked SOCE (Fig. 1D). It might seem surprising that so many combinations of four different *itpr* mutants should inhibit SOCE. However, selection of the original mutant combinations was based on flight phenotypes (Banerjee et al., 2004), and restoring SOCE can rescue these flight defects (Agrawal et al., 2010). The original selection might therefore have preferentially identified *itpr* mutants that attenuate SOCE. Immunoblots established that expression of IP_3_R, STIM and Orai were similar in the larval central nervous system from wild-type and *itpr* mutant flies (Fig. S1B,C).

Similar functional consequences of mutant *itpr* were observed in cultured haemocytes from *Drosophila* larvae, where *itpr* mutants attenuated thapsigargin-evoked SOCE without affecting basal [Ca^2+^]_c_ or Ca^2+^ release from intracellular stores (Fig. S2).

The results so far establish that loss of IP_3_R or mutations within IP_3_R attenuate SOCE without affecting the Ca^2+^ content of the intracellular stores. The effects are not due to loss of STIM or Orai.

### Over-expressed STIM and Orai restores SOCE in neurons with mutant IP_3_Rs

We used the mutant *itpr* combination *itpr*^S224F/G1891S^ to examine the effects of overexpressing STIM and Orai on SOCE in cultured neurons. We chose this combination because it has been the most extensively studied of the heteroallelic mutant *itpr* combinations (Agrawal et al., 2010; Venkiteswaran and Hasan, 2009). The response to thapsigargin in Ca^2+^-free medium was unaffected by overexpression of STIM and Orai (Fig. 1C,E). Rates of recovery from these [Ca^2+^]_c_ increases were also unaffected (Fig. 1F). These results demonstrate that the ER Ca^2+^ content, passive leak of Ca^2+^ from the ER, and rates of Ca^2+^ extrusion from the cytosol were similar in neurons with mutant or wild-type IP_3_R, and unaffected by overexpression of STIM and Orai. However, SOCE in neurons expressing mutant IP_3_R was restored by overexpression of STIM with Orai (Fig. 1D,E). This is consistent with behavioural analyses (Fig. S3A,B) (Agrawal et al., 2010). A similar restoration of SOCE upon overexpression of STIM has been reported in neurons in which IP_3_R expression was reduced by small interfering RNA (siRNA) (Deb et al., 2016). Hence, even though mutant IP_3_Rs do not affect expression of STIM or Orai, the attenuated SOCE can be compensated for by overexpressing STIM and Orai (Fig. 1D). The results so far suggest that the IP_3_R regulates SOCE downstream of ER Ca^2+^ release, perhaps by influencing interactions between STIM and Orai.

### Mutant IP_3_Rs attenuate the association of STIM with Orai after store depletion

**Fig. 2.**
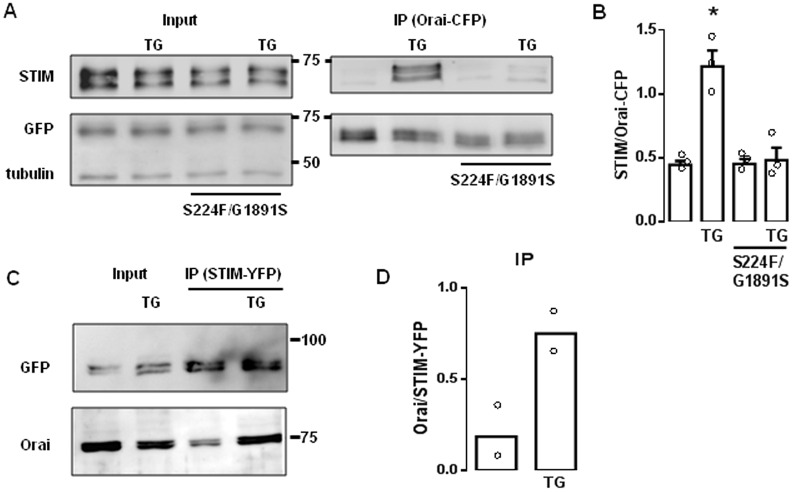
**Mutant IP_3_Rs attenuate association of STIM and Orai after store depletion.** (A) Western blots from brains of larval *Drosophila* expressing Orai–CFP with WT or mutant IP_3_Rs, and treated with thapsigargin (TG, 10 µM in Ca^2+^-free HBM for 10 min) as indicated. The input lysates (equivalent to 20% of the immunoprecipitated sample) and anti-GFP immunoprecipitates (IP) are shown. α-tubulin provides a loading control. The positions of molecular mass markers (kDa) are shown between blots. (B) Summary results for the ratio of the intensities of the STIM to Orai–CFP bands (mean±s.e.m., *n*=3). *P<0.05, paired Student's *t*-test relative to the respective control. (C) WT brains expressing STIM–YFP show results of immunoprecipitation (with anti-GFP antibody) after treatment with thapsigargin as indicated. The lysate lanes contain the equivalent of 20% of the immunoprecipitated sample lanes. (D) Summary results show the ratio of the intensities of the Orai to STIM–YFP bands (mean and individual values are shown; *n*=2).

We tested whether IP_3_R mutations affect interactions between STIM and Orai using an ectopically expressed *dOrai-CFP^+^* transgene with a pan-neuronal driver (*Elav^C155^*). This allowed immunoprecipitation of Orai with an anti-GFP antibody. Expression of Orai–CFP did not restore SOCE in *itpr* mutant neurons (Fig. S3C,D). Treatment with thapsigargin enhanced the pulldown of STIM with anti-GFP antibody from lysates of wild-type brain, consistent with enhanced interaction between STIM and Orai after store depletion. However, the pulldown of STIM from thapsigargin-treated brains with mutant IP_3_R was much reduced (Fig. 2A,B). In the reciprocal immunoprecipitation using wild-type brain expressing STIM–YFP, thapsigargin increased the pulldown of Orai with the anti-GFP antibody (Fig. 2C,D). There was no detectable IP_3_R in this immunoprecipitate (data not shown), suggesting that any interaction between IP_3_R and STIM or Orai, whether direct or through other proteins, might be too weak to survive immunoprecipitation. It was impracticable to assess the effects of mutant IP_3_R in these immunoprecipitation experiments because STIM–YFP rescued the mutant IP_3_R phenotypes (Fig. 1D) (Agrawal et al., 2010). These results suggest that wild-type IP_3_R stabilizes interactions between STIM and Orai after depletion of Ca^2+^ stores.

**Fig. 3.**
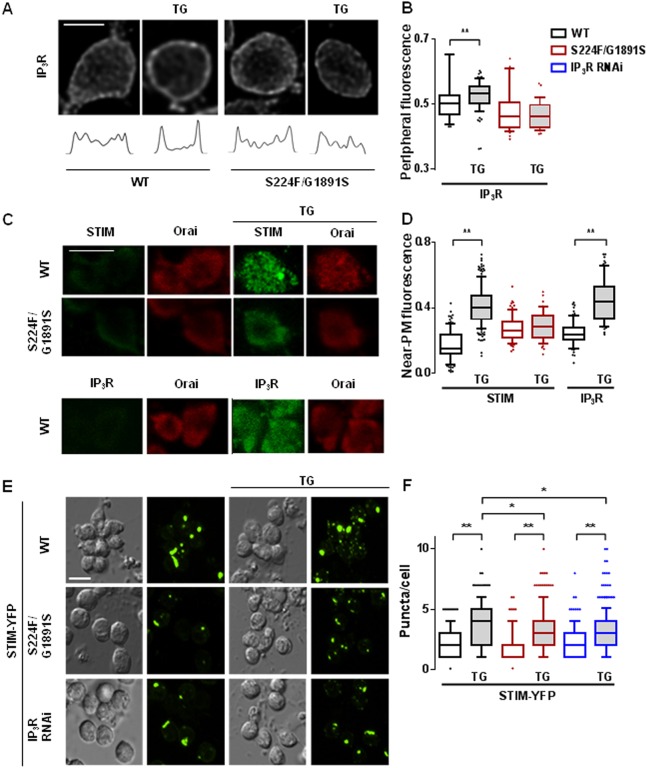
**Mutant IP_3_Rs attenuate translocation of IP_3_R and STIM after store depletion.** (A) Typical confocal images across the mid-plane of fixed neurons immunostained for IP_3_R after treatment with thapsigargin (TG, 10 µM in Ca^2+^-free HBM for 10 min). Fluorescence profiles are shown below each image. (B) Summary results for peripheral IP_3_R immunostaining as a fraction of total cellular fluorescence for the indicated genotypes. The same colour key applies to panels B, D and F. (C) Optical section at the plasma membrane of neurons expressing mutant (*itpr*^S224F/G1891S^) or WT IP_3_R with and without thapsigargin-treatment showing immunostaining for Orai and STIM or IP_3_R. (D) Summary results for near-plasma-membrane STIM and IP_3_R labelling (near-plasma membrane/total). (E) Differential interference contrast (DIC) and optical section of GFP fluorescence at plasma membrane of neurons expressing STIM–YFP with mutant (*itpr*^S224F/G1891S^) or after treatment with siRNA to IP_3_R. The effects of treatment with thapsigargin are shown. (F) Summary results for the number of STIM–YFP puncta/cell. In B, D and F, the box represents the 25–75th percentiles, and the median is indicated. The whiskers show the 10–90th percentiles. Outliers are represented by dots. **P<0.01, *P<0.05, Kruskal–Wallis test, followed by Wilcoxon signed-rank post-hoc test [>50 cells from at least five independent experiments (B,D); >200 cells from at least five independent experiments (F)]. Scale bars: 5 µm.

To avoid reversal of attenuated SOCE in neurons with mutant IP_3_Rs after overexpression of STIM (Fig. 1D), we used immunostaining of fixed neurons to examine the effects of store depletion on the distribution of endogenous STIM, Orai and IP_3_R. We quantified the near-plasma-membrane distribution of STIM and IP_3_R by measuring either peripheral fluorescence in confocal sections across a mid-plane of the cell (Fig. 3A,B; Movies 1–4) or total fluorescence within a plane that included mostly plasma membrane (Fig. 3C,D) (see Materials and Methods). In wild-type neurons, thapsigargin significantly increased the amount of STIM detected near the plasma membrane. This redistribution of STIM was attenuated in neurons with mutant IP_3_R (*itpr*^S224F/G1891S^) (Fig. 3C,D). Store depletion also increased the intensity of IP_3_R immunostaining near the plasma membrane (Fig. 3A–D). There was no significant redistribution of IP_3_R in thapsigargin-treated neurons expressing mutant IP_3_R (Fig. 3A,B). Thapsigargin stimulated formation of STIM puncta in neurons expressing STIM–YFP (Fig. 3E), although the puncta were not detected with endogenous STIM. This is consistent with the effects of store depletion in mammalian cells, where STIM puncta are typically observed after overexpression of tagged STIM. The formation of STIM–YFP puncta after store depletion was significantly attenuated in *Drosophila* neurons with mutant IP_3_Rs (*itpr*^S224F/G1891S^); and siRNA for the IP_3_R appeared to have a similar effect (Fig. 3E,F). The translocation of STIM and wild-type IP_3_R towards the plasma membrane after store depletion was not due to a general reorganization of the ER because co-staining of neurons for STIM and another ER protein (GFP-tagged protein disulphide isomerase, PDI–GFP) revealed that only STIM redistributed after thapsigargin treatment (Fig. S4). These results demonstrate that IP_3_R and STIM accumulate in peripheral ER near the plasma membrane after store depletion, and loss of IP_3_R or mutations within it inhibits the translocation of STIM.

### Mutant IP_3_Rs attenuate formation of Orai puncta at the plasma membrane

**Fig. 4.**
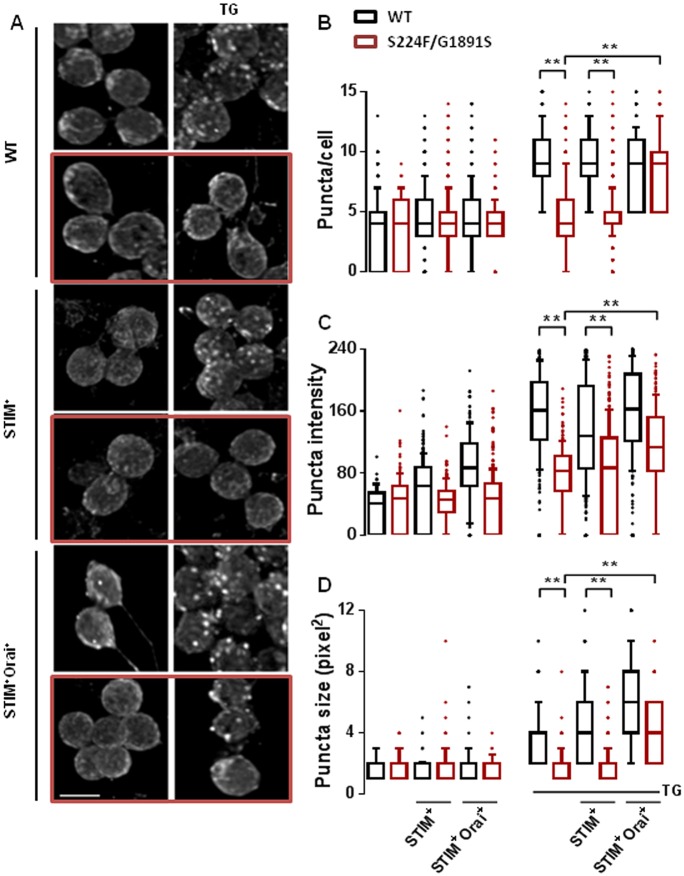
**Mutant IP_3_Rs attenuate clustering of Orai after store depletion.** (A) Typical confocal images from neurons with or without thapsigargin (TG) treatment and immunostained for endogenous Orai. The effects of the mutant IP_3_R combination (S224F/G1891S, red boxes), and overexpression of STIM alone (STIM^+^) or with Orai (STIM^+^Orai^+^) are shown. Scale bar: 5 µm. The representative confocal images show *z*-stacks of the deconvoluted sections. Each section was analysed individually for the summary analyses. (B–D) Summary data for the number of puncta/cell (B), intensity of fluorescence within puncta (C) and size of puncta (D). The colour key applies to all three panels. In B–D, the box represents the 25–75th percentiles, and the median is indicated. The whiskers show the 10–90th percentiles. Outliers are represented by dots. **P<0.01, Kruskal–Wallis test, followed by Wilcoxon signed-rank post-hoc test (>150 cells from at least three independent experiments).

Using an antibody to endogenous Orai, we observed that depletion of Ca^2+^ stores with thapsigargin stimulated formation of Orai puncta in neurons expressing wild-type IP_3_R, but not in neurons expressing mutant IP_3_Rs (*itpr^S224F/G1891S^*) (Fig. 4A). Furthermore, the sparse Orai puncta that did form in neurons with mutant IP_3_Rs were both smaller and less intensely stained than in neurons with wild-type IP_3_Rs (Fig. 4B–D). Overexpression of STIM had no effect on the formation of Orai puncta in neurons with either genotype, although it partially restored SOCE (Fig. 1D). However, after overexpression of both STIM and Orai, store depletion stimulated formation of Orai puncta that were similar in neurons with wild-type or mutant IP_3_Rs (Fig. 4B–D). The ability of overexpressed STIM and Orai to rescue formation of Orai puncta in neurons with mutant IP_3_Rs coincides with a similar rescue of SOCE in mutant neurons (Fig. 1D) and of flight in flies with mutant IP_3_R (Fig. S3A,B). These results suggest that overexpression of STIM with Orai can override the requirement of IP_3_R for formation of Orai puncta or SOCE after store depletion. However, when STIM and Orai are expressed at native levels in *Drosophila*, the interaction between them, the formation of Orai puncta and the activation of SOCE are enhanced by IP_3_R.

## DISCUSSION

**Fig. 5.**
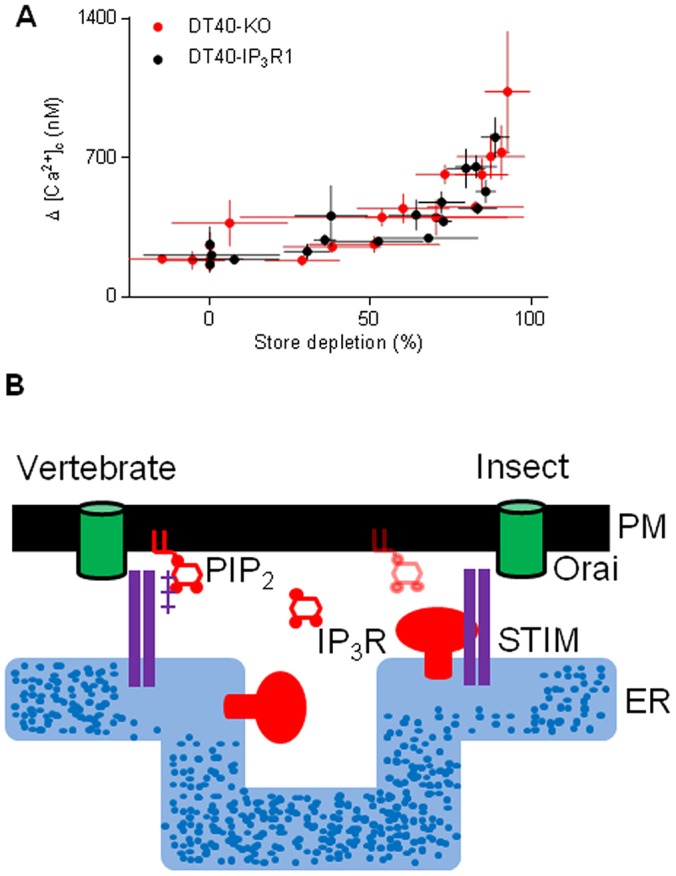
**Coordination of store depletion and SOCE in insects and vertebrates.** (A) DT40-KO or DT40-IP_3_R1 cells were incubated with different concentrations of a reversible inhibitor of the ER Ca^2+^ pump, cyclopiazonic acid (CPA, 0.1–3 µM) for 15 min in Ca^2+^-free HBS. In parallel wells, the peak increase in [Ca^2+^]_c_ evoked by ionomycin (1 µM, to determine the Ca^2+^ content of the intracellular stores) or restoration of extracellular Ca^2+^ (1.5 mM, to determine SOCE) were measured. Results (means±s.e.m., *n*=3, with three replicates in each) show the relationship between store depletion and SOCE for the two cell lines. (B) Substantial loss of Ca^2+^ from the ER causes STIM to cluster and assemble with Orai at ER–plasma-membrane junctions. The polybasic cytoplasmic tail of vertebrate STIM1 binds to PIP_2_ within the plasma membrane and contributes to its targeting to junctions. *Drosophila* STIM lacks a PIP_2_-binding motif, but, after store-depletion, *Drosophila* STIM moves to ER–plasma-membrane junctions, and so too does IP_3_R where it might bind to PIP_2_. Physiological stimuli, through IP_3_, probably trigger the large decrease in luminal [Ca^2+^] needed to activate STIM1 in only a subset of the ER. Targeting of vertebrate STIM1 to plasma membrane enriched in the PIP_2_, from which IP_3_ is synthesized, ensures that the machinery needed to locally deplete Ca^2+^ stores remains closely associated with essential components of the SOCE pathway. We speculate that in *Drosophila*, association of IP_3_R with STIM and Orai at ER–plasma-membrane junctions might fulfil a similar role.

Before STIM was identified as the Ca^2+^ sensor that regulates SOCE, IP_3_Rs were speculated to adopt this role (Irvine, 1990). However, thapsigargin evokes SOCE in avian DT40 cells lacking IP_3_Rs (Sugawara et al., 1997) (Fig. 5A) and SOCE can be functionally reconstituted with Orai and STIM (Zhou et al., 2010). IP_3_Rs are not, therefore, essential for empty Ca^2+^ stores to activate SOCE. Our results, showing that SOCE is attenuated in *Drosophila* neurons with mutant IP_3_Rs (Fig. 1), suggest that IP_3_Rs can modulate SOCE. Depletion of intracellular Ca^2+^ stores caused STIM and mutant IP_3_Rs to accumulate near the plasma membrane (Fig. 3A–F), STIM and Orai to associate (Fig. 2), formation of STIM and Orai puncta (Figs 3 and 4), and activation of SOCE (Fig. 1). These responses were attenuated in neurons with mutant IP_3_Rs. The effects of mutant IP_3_Rs were not due to a dominant-negative property of the mutants because SOCE was also attenuated when IP_3_Rs expression was reduced by siRNA (Fig. 1D) (Agrawal et al., 2010) and the mutant IP_3_Rs reduced SOCE only when both alleles were mutated (Fig. 1D). We suggest that after store-depletion, both STIM and IP_3_R translocate to ER–plasma-membrane junctions, where IP_3_R might stabilize the interaction of STIM with Orai, and thereby promote SOCE (Fig. 5B).

In some mammalian cells, IP_3_Rs have been shown to colocalize with Orai1 (Lur et al., 2011) and to interact with STIM1, Orai1 and transient receptor potential canonical channels (TRPCs) (Hong et al., 2011), but there is no functional evidence that IP_3_Rs directly contribute to SOCE mediated by Orai. Block of SOCE by an antagonist of IP_3_Rs, 2-aminoethoxydiphenyl borate (2-APB), was originally suggested to reflect IP_3_R coupling to a SOCE channel (Ma et al., 2000), but it is now attributed to direct inhibition of STIM and Orai by 2-APB. However, most analyses of SOCE use thapsigargin to completely deplete intracellular Ca^2+^ stores, and overexpressed proteins to track movements of Orai and STIM. These exaggerated conditions successfully identify key features of SOCE, but they might override more subtle modulatory influences, including, for example, the contribution of PIP_2_ to recruitment of STIM1 to ER–plasma-membrane junctions (Hogan, 2015; Park et al., 2009). We used DT40 cells with and without IP_3_R1 (encoded by *itpr1*) and examined SOCE after graded depletion of intracellular Ca^2+^ stores to assess whether partially depleted stores might more effectively activate SOCE in the presence of IP_3_R. However, the relationship between store depletion and SOCE was unaffected by expression of IP_3_R1 (Fig. 5A). Hence, there is no compelling evidence to suggest that the contribution of IP_3_R to SOCE in *Drosophila* is a feature shared with vertebrates.

Inhibition of Orai clustering in *Drosophila* neurons with mutant IP_3_Rs is reminiscent of the effects of septin depletion in mammalian cells. Septin 4 concentrates PIP_2_ around Orai1 and facilitates recruitment of STIM1 through its polybasic cytoplasmic tail (Sharma et al., 2013). Assembly of STIM–Orai complexes at PIP_2_-enriched plasma membrane domains concentrates the complexes at regions best equipped to sustain production of the IP_3_ that evokes Ca^2+^ release from stores. Such colocalization of Ca^2+^ release and SOCE might be important because activation of SOCE by physiological stimuli probably requires substantial local depletion of intracellular Ca^2+^ stores (Bird et al., 2009; Luik et al., 2008). However, *Drosophila* STIM lacks the polybasic tail through which mammalian STIM1 binds to PIP_2_ (Huang et al., 2006). Association of *Drosophila* STIM with IP_3_R, which might itself bind to PIP_2_ (Lupu et al., 1998), could serve a function analogous to PIP_2_-mediated targeting of STIM1 in mammals. Recent evidence demonstrating a link between Septin 7, IP_3_R and SOCE (Deb et al., 2016) suggests that IP_3_R might influence STIM–Orai interactions within a larger macromolecular complex. We speculate that interaction of mammalian STIM1 with PIP_2_ might ensure that intracellular stores locally depleted of Ca^2+^ by IP_3_ are effectively localized to STIM–Orai complexes (Fig. 5B). Translocation of both IP_3_R and STIM to ER–plasma-membrane junctions after store depletion, where IP_3_R facilitates the interaction of STIM with Orai, might fulfil a similar role in *Drosophila* (Fig. 5B).

## MATERIALS AND METHODS

### *Drosophila* strains

Single-point mutants of the *itpr* gene were characterized as described previously (Joshi et al., 2004; Srikanth et al., 2004). *UAS* transgenic strains were generated by injecting *Drosophila* embryos with a *pUAST* construct. All fly strains, including *Elav^C155^GAL4* (pan neuronal, Bloomington Stock Center, Indiana University, Bloomington, IN), and RNAi lines for *itpr* (no. 1063, National Institute of Genetics, Japan), *STIM* (no. 47073) and *Orai* (no. 12221) were procured from the Vienna *Drosophila* Resource Centre, Austria. The Canton-S strain was used as the wild-type control.

### Measurements of [Ca^2+^]_c_ in primary cultures of *Drosophila* neurons

Materials, unless stated otherwise, were from ThermoFisher Scientific (Waltham, MA). Methods for primary cultures were adapted from Wu et al. (1983). The brain and ventral ganglia from *Drosophila* third-instar larvae were dissociated by incubation for 20 min at 25°C in Schneider's medium containing collagenase (0.75 µg/µl) and dispase (0.4 µg/µl, Roche, Burgess Hill, UK). After centrifugation (600 ***g*** for 5 min), cells were plated onto poly-l-lysine-coated coverslips in HEPES-buffered medium [HBM, in mM: HEPES (30), NaCl (150), KCl (5), MgCl_2_ (1), CaCl_2_ (1), sucrose (35), pH 7.2] or (for most experiments) Dulbecco's modified Eagle's medium (DMEM) with F12 and Glutamax-I, NaHCO_3_ and sodium pyruvate, and supplemented with 20 mM HEPES (pH 7.2) and 10% fetal bovine serum. This enriched medium substantially reduced the heterogeneity of the Ca^2+^ signals between neurons. All culture media contained 50 units/ml penicillin, 50 µg/ml streptomycin and 10 µg/ml amphotericin B. Cells were incubated at 25°C in humidified air with 5% CO_2_. After 14–16 h, cells were loaded with fluo 4 by incubation at 25°C for 30 min with fluo 4-AM (2.5 µM) and Pluronic F-127 (0.02%) in HBM. Medium was then replaced with HBM, and after a further 10–30 min, with Ca^2+^-free HBM containing 0.5 mM EGTA. Cells were immediately imaged at 15-s intervals with excitation at 488 nm and emission at 520 nm using a Nikon TE2000 microscope with a 60×1.4 NA objective, Evolution QEi CCD camera and QED imaging software (Media Cybernetics, Rockville, MD). Background fluorescence (measured from an area without cells) was subtracted from all measurements before calculation of ΔF/F_0_, where F_0_ is the initial fluorescence and ΔF is the difference between basal and peak fluorescence.

### Measurements of SOCE in DT40 cells

Avian DT40 cells in which endogenous IP_3_R genes are disrupted (DT40-KO cells) (Sugawara et al., 1997) or the same cells stably expressing rat IP_3_R1 (DT40-IP_3_R1) were used to determine the contribution of IP_3_R to SOCE in cells from vertebrates. DT40 cells (10^7^ cells/ml) were loaded with fluo-4 by incubation at 20°C with fluo-4 AM (2 µM) in HBS containing BSA (1 mg/ml) and Pluronic F-127 (0.02% w/v) [HBS in mM: NaCl (135), KCl (5.8), MgCl_2_ (1.2), CaCl_2_ (1.5), HEPES (11.6), d-glucose (11.5) pH 7.3]. After 60 min, cells were centrifuged (650 ***g***, 2 min), re-suspended in HBS (5×10^6^ cells/ml) and distributed (50 µl/well) into poly-l-lysine-coated half-area 96-well plates. After centrifugation (300 ***g***, 2 min) fluorescence (excitation 485 nm, emission 525 nm) was recorded at 1.44-s intervals at 20°C in a FlexStation 3 plate-reader. Fluorescence signals (F) were calibrated to [Ca^2+^]_c_ from:
(1)
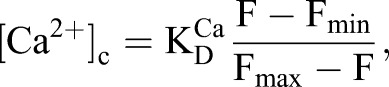
where, F_min_ and F_max_ are the fluorescence values determined in parallel wells by addition of Triton X-100 (0.1% w/v) and either BAPTA (10 mM) for F_min_, or CaCl_2_ (10 mM) for F_max_, and K_D_^Ca^=345 nM.

### Immunoprecipitation, western blotting and immunocytochemistry

For immunoprecipitation analyses, neuronal cultures were stimulated, washed and lysed in cold PBS (pH 7.4) containing 1% NP-40 and 5 mM EDTA, Roche protease inhibitor tablet and 10 µM MG-132. The lysate was homogenized by passage through a 26 G needle, mixed (30 min, 4°C) and after centrifugation (14,000 ***g***, 20 min), the supernatant (0.5 µg protein/µl in PBS) was incubated with Dynabeads (2 mg) bound to anti-GFP antibody (15 µg, #A-11122). After 18–20 h at 4°C, the beads were washed and lysed according to the manufacturer's protocol, and used for western blotting. The antibodies used were: *Drosophila* STIM (1:10; Abexome, Bangalore, India) (Agrawal et al., 2010), GFP (1:5000; #SC9996, Santa Cruz Biotechnology, Dallas, TX) and α-tubulin (1:5000; #E7, Developmental Studies Hybridoma Bank, University of Iowa, IA). HRP-conjugated anti-rabbit-IgG (#32260; Thermo Scientific), anti-mouse-IgG (#7076S; Cell Signaling Technologies, Danvers, MA) and anti-rat-IgG (#012030003; Jackson ImmunoResearch, West Grove, PA) secondary antibodies were used and visualized with SuperSignal West Dura Extended Duration Substrate. For immunostaining, methods were adapted from Wegener et al. (2004). Cultured neurons were treated with thapsigargin and then fixed (30 min, 25°C, 3.5% paraformaldehyde and 0.5% glutaraldehyde in PBS), washed three times (PBS with 0.5% BSA, 0.05% Triton X-100 and 0.05% glycerol) and permeabilized (1 h, PBS with 5% BSA, 0.5% Triton X-100, 0.5% glycerol). Cells were incubated for 12 h with primary antibody [rabbit for *Drosophila* IP_3_R (1:300) (Srikanth et al., 2004), mouse for *Drosophila* STIM (1:10) (Agrawal et al., 2010) and rat for *Drosophila* Orai (1:1000) (Pathak et al., 2015)]. Cells were then washed, incubated (30 min, 4°C) with appropriate secondary antibody (1:500) conjugated to Alexa Fluor 488 (#A1108; Thermo Scientific), Alexa Fluor 594 (#20185) or Alexa Fluor 633 (#A201948) and washed. Images were acquired using an Olympus laser-scanning FV1000 SPD confocal microscope with 60×1.3 NA oil immersion objective. All images were corrected for background by subtraction of fluorescence recorded outside the cell.

### Image analysis

Confocal images were deconvoluted using Huygens 4.5 software (SVI, The Netherlands) as described previously (Deb et al., 2016). To quantify the peripheral fluorescence of immunostained IP_3_R in each neuron, most of which have a near-circular profile (Fig. 3A), an automated algorithm (Matlab) was used to identify the confocal section with the maximal perimeter. Within this section, which we describe as the ‘mid-section’, the centre of the cell was identified and an average radius calculated (r). Fluorescence intensities were then calculated for the central circular region (with r/2) and for the remaining peripheral annulus. The ratio (peripheral fluorescence to total fluorescence) was then used to report the redistribution of IP_3_R.

To quantify near-plasma-membrane immunostaining of STIM and IP_3_R, we used Orai immunostaining to manually identify the confocal section within which most plasma membrane apposed the coverslip (Fig. 3C). The fluorescence intensity within this optical section relative to that from the entire cell was used to report the near-plasma-membrane distribution.

To quantify the distribution of STIM–YFP (Fig. 3G,H) and Orai (Fig. 4) puncta, every confocal section (∼15 sections/cell) was analysed (Deb et al., 2016). Puncta were identified automatically (Matlab) as fluorescence spots that exceeded average cellular fluorescence by at least 1.9× the s.d. and occupied a square with sides of 1–12 pixels (1 pixel=103 nm×103 nm) with a circularity of 0–0.3. Analysis of sequential sections within the *z*-stack allowed non-redundant counting of puncta, from which the total number of puncta/cell was determined. The section in which a punctum had the brightest intensity was used for analysis, and then normalized to the mean intensity of Orai for the cell.

### Statistical analysis

Most data were analysed using non-parametric methods (Kruskal–Wallis test for variance followed by Wilcoxon signed-rank post-hoc tests). These data are presented as box and whisker plots showing medians, 25–75th percentiles (boxes), 10–90th percentiles (whiskers) and points for values beyond the 10th and 90th percentiles. Student's *t*-tests were used for statistical analysis of western blots (Fig. 2B) and Ca^2+^ signals in DT40 cells (Fig. 5A).
